# Sperm-Mediated Transgenerational Inheritance

**DOI:** 10.3389/fmicb.2017.02401

**Published:** 2017-12-04

**Authors:** Corrado Spadafora

**Affiliations:** Institute of Translational Pharmacology, National Research Council of Italy, Rome, Italy

**Keywords:** spermatozoa, LINE-1 retrotransposons, reverse transcriptase, exosomes, transgenerational inheritance, evolution

## Abstract

Spermatozoa of virtually all species can spontaneously take up exogenous DNA or RNA molecules and internalize them into nuclei. In this article I review evidence for a key role of a reverse transcriptase (RT) activity, encoded by LINE-1 retrotransposons, in the fate of the internalized nucleic acid molecules and their implication in transgenerational inheritance. LINE-1-derived RT, present in sperm heads, can reverse-transcribe the internalized molecules in cDNA copies: exogenous RNA is reverse-transcribed in a one-step reaction, whereas DNA is first transcribed into RNA and subsequently reverse-transcribed. Both RNA and cDNA molecules can be delivered from sperm cells to oocytes at fertilization, further propagated throughout embryogenesis and inherited in a non-Mendelian fashion in tissues of adult animals. The reverse-transcribed sequences are extrachromosomal, low-abundance, and mosaic distributed in tissues of adult individuals, where they are variably expressed. These “retrogenes” are transcriptionally competent and induce novel phenotypic traits in animals. Growing evidence indicate that cancer tissues produce DNA- and RNA-containing exosomes. We recently found that these exosomes are released in the bloodstream and eventually taken up into epididymal spermatozoa, consistent with the emerging view that a transgenerational flow of extrachromosomal RNA connects soma to germline and, further, to next generation embryos. Spermatozoa play a crucial bridging role in this process: they act as collectors of somatic information and as delivering vectors to the next generation. On the whole, this phenomenon is compatible with a Lamarckian-type view and closely resembles Darwinian pangenesis.

## Spermatozoa As A Source of Reverse Transcriptase-Mediated Extrachromosomal Information: A Look to the Past

It is a well-established notion that mature spermatozoa have the spontaneous ability to take up exogenous DNA molecules and to internalize them in their nuclei (reviewed by Spadafora, 1998). This permeability is a distinctive feature of spermatozoa, both epididymal and ejaculated (after wash-off of seminal fluid), from virtually all animal species including humans (Smith and Spadafora, 2005). Thus, in spite of the highly compact and impenetrable structure of their nuclei, sperm cells are in fact highly permeable to foreign molecule intrusion. Intense investigations of this phenomenon revealed that the interaction of exogenous DNA molecules with sperm cells, as well as their subsequent nuclear internalization, are well-regulated processes mediated by a network of specific factors (Spadafora, 1998). Parallel studies have revealed that spermatozoa can also take up RNA molecules and internalize them in their nuclei. Somewhat unexpectedly, these RNAs are reverse-transcribed into cDNA copies by a biologically active reverse transcriptase (RT) activity encoded by LINE-1 retrotransposons and present in sperm nuclei (Giordano et al., 2000; Spadafora, 2008). The LINE-1-derived RT interplays with a DNA-dependent RNA polymerase, also present in spermatozoa (Fuster et al., 1977), which together amplify the cDNAs copy number, mimicking a “natural” PCR/RT-PCR process. Most newly generated cDNA copies are released from spermatozoa into the medium and can be taken up again by further spermatozoa and internalized in their nuclei. Through this continuously cycling process, cDNA copies are evenly distributed among the vast majority of sperm cells suspension. Work with murine models showed that the RT-generated cDNAs are: delivered to oocytes at fertilization (Giordano et al., 2000; Pittoggi et al., 2006), maintained as low-copy number (below one copy/genome) non-integrated extrachromosomal sequences throughout development, mosaic propagated in the tissues of founder individuals, eventually transmitted in a non-Mendelian fashion to the next generation, transcriptionally competent and able to generate phenotypic variations in animals of both generations (Sciamanna et al., 2003; Pittoggi et al., 2006). These results suggest that spermatozoa provide a previously unrecognized source of RT-mediated information, not linked to chromosomal genes, and, at the same time, act as propagating vectors throughout generations.

These findings raise several puzzling questions. First, does the ability of sperm cells to take up foreign nucleic acid molecules reflects an enforced behavior when they come in contact with RNA under conditions of *in vitro* assays, or else do spermatozoa naturally collect and carry foreign molecules under physiological conditions *in vivo*? Second, does the RT activity stored in spermatozoa represent a functionless remnant of ancestral retrotransposon activity, brought to new life in response to occasional intrusions of foreign molecules, or does it exert an extant physiological role in development? These two issues, i.e., the sperm permeability to exogenous RNA, and the sperm RT that uses the latter as a substrate for retrotranscription, raise the third key question of whether these phenomena are physiologically relevant or, in other words, whether they occur in nature to generate a source of novel information. To begin to address these issues, it was imperative to characterize the RNA population stored in spermatozoa and possibly identify its origin. In recent years, high-throughput technologies and next generation sequence analysis have revealed a highly complex composition of spermatozoal RNA, whose components are increasingly emerging as key players in epigenetic inheritance processes, as will be seen in more depth in the following paragraphs.

## The Complex Transcriptional Landscape of Mature Spermatozoa

Traditional views considered spermatozoa as transcriptionally silent cells (Grunewald et al., 2005) and sperm RNAs as negligible remnants produced during spermatogenesis. More recent data however contrast with these views, showing that mature spermatozoa in fact contain a complex population of coding RNAs, small non-coding RNA classes, and, finally, LINE-1, SINE/Alu, and LTR repeat-associated transcripts (Jodar et al., 2013; Sendler et al., 2013; Miller, 2014). Small non-coding RNAs account for a considerable proportion of spermatozoal RNA (Krawetz et al., 2011; Kawano et al., 2012), mainly represented by piRNAs produced during spermatogenesis, tsRNA (tRNA-derived), and to a lesser extent, microRNAs (miRNAs) are instead predominant in epididymal spermatozoa (Chen et al., 2016b). Importantly, the composition of the spermatozoal RNA population varies in response to paternal exposure to a variety of stressing conditions (Rodgers et al., 2013; Brieno-Enriquez et al., 2015), a circumstance that can have crucial consequences for the fate and health of the progeny. Most importantly, growing data are revealing that RNAs of somatic origin also contribute to the composition of the sperm RNA cargo in the form of selectively retained RNAs derived from soma-to-spermatozoa intercellular communication. This flow is mediated by a special class of epididymis-derived nanovesicles, called epididymosomes, which shuttle miRNAs and tRNA fragments from the epididymal tissue to mature sperm cells (Belleannée et al., 2013; Vojtech et al., 2014; Sharma et al., 2016). The shuttled sperm RNA, containing several 100s of developmentally relevant small RNAs, are the product of a “sieving” process, as their profiles are distinct from those of the surrounding soma (Reilly et al., 2016). The modulation of the sperm RNA content occurs during maturation of spermatozoa between the proximal and distal epididymal segments, and identifies the epididymis as a key site for the establishment of the sperm epigenome (Nixon et al., 2015).

We recently reported that epididymal spermatozoa can incorporate RNA from somatic cell-released exosomes: indeed, we found that human melanoma cells, engineered to express EGFP and inoculated in nude mice, release EGFP RNA-containing nanovesicles in the bloodstream of the animals; a proportion of that RNA reaches the epididymis and becomes internalized in sperm heads (Cossetti et al., 2014). This finding shows that the flow of RNA delivered to spermatozoa originates not only from the surrounding epididymal soma, but also from distant, unrelated districts of the body. Nanovesicles act as the ideal vectors of such delivery. Sperm heads are the final recipients of this extrachromosomal information due to their ability to spontaneously take up exogenous molecules, as mentioned above. On the whole, these data indicate that the impenetrable Weismann barrier, considered for a long time as a cornerstone of modern genetics, can in fact be breached by nanovesicle-mediated flows of extrachromosomal RNA (Eaton et al., 2015).

## Breaking the Weismann Barrier: A Sperm-Mediated RNA-Based Flow Connects Soma to the Next Generation Embryos

In a seminal article, Krawetz and collaborators (Ostermeier et al., 2004) first reported that the sperm-specific RNA cargo is delivered to oocytes at fertilization. That finding proved that not only the male genome, but also extrachromosomal RNA carried by sperms, contribute to the zygote formation. However, the sperm RNA *per se* is not strictly required for embryonic development, as parthenogenetic mice can be successfully generated by microinjecting haploid, or bimaternal embryonic stem cells in murine oocytes (Li et al., 2016; Zhong et al., 2016). The latter finding indicates that all the fundamental information to support the developmental program, from fertilization to adulthood, is linked to chromosomal genes.

A novel turn to the field is being provided by recent data indicating that the composition of sperm RNA reflects the lifestyle habits and carry the “memory” of paternal experiences; that RNA-based memory is transmissible to the offspring as paternally acquired characteristics, with the potential to affect the health and overall biological fate of the progeny (reviewed by Liebers et al., 2014; Klosin and Lehner, 2016). Of remarkable interest are recent experiments that have assessed the potential of sperm RNAs as transgenerational modifiers in response to parental environmental or stressing conditions (Carone et al., 2010; Rodgers et al., 2013, 2015), including diet (Fullston et al., 2013; Chen et al., 2016a; Huypens et al., 2016), cigarette smoke (Marczylo et al., 2012), odor sensitivity (Dias and Ressler, 2014), and cognitive and behavioral conditioning (Rodgers et al., 2013; Gapp et al., 2014). RNA was unambiguously identified as the transgenerational modifier in a large set of compelling experimental data, showing that offspring generated from normal zygotes microinjected with sperm RNA recapitulate the phenotypical traits of the RNA donor animals (Rassoulzadegan et al., 2006; Gapp et al., 2014; Grandjean et al., 2015; Chen et al., 2016a).

Together, these data show that inheritance is not exclusively linked to chromosomal genes. Indeed, a subtle yet effective flow of RNA is established between somatic tissues and the next generation embryos. Spermatozoa are the pivots, playing a dual role both as collectors of paternal extrachromosomal RNA and as their vectors to the offspring. The emerging evidence that RNA-based information can travel from soma to germline subvert the Weismann’s theory and provide a foundation for the inheritance of acquired traits with far reaching implications for evolutionary processes.

## RT Encoded By Line-1 Retrotransposons As Modulator of Early Embryonic Development

In addition to being stored in mature spermatozoa, LINE-1-encoded RT is also abundantly expressed in early embryos and is implicated in the genesis and propagation of extrachromosomal information. We have found that LINE-1 retrotransposon-encoded RT is triggered soon after fertilization in both zygotic pronuclei, predominantly in the paternal pronucleus (Vitullo et al., 2012), and remains active in early preimplantation embryos (Pittoggi et al., 2003). RT plays a crucial role in early development: indeed, RT inhibition, induced either by pharmacological RT inhibitors (Pittoggi et al., 2003), or by downregulating LINE-1 expression by microinjecting antisense oligonucleotides in zygotic pronuclei (Beraldi et al., 2006), causes a drastic arrest of embryo development at the 2- or 4-cell stages. These results suggest that RT is strictly necessary for the unfolding of the developmental program from the second cell division, as the first cleavage exploits the maternal RNA stored in oocytes (Tang et al., 2007).

Although neither the specific role(s) nor the mechanism of action of embryonic RT are yet fully clarified, emerging data suggest that RT controls the biogenesis of miRNAs, a class of RNA that is globally, yet transiently, suppressed in early embryogenesis (Suh et al., 2010), concomitant with the up-regulation of RT expression (Pittoggi et al., 2003; Vitullo et al., 2012).

The link between LINE-1-encoded RT and miRNA biogenesis has been investigated in some depth in cancer cells. In striking analogy with early embryos, RT is also highly expressed in most cancer types from very early stages (reviewed by Sinibaldi-Vallebona et al., 2011; De Luca et al., 2016). In parallel with high RT activity, the biogenesis of LINE-1-derived miRNAs (Lu et al., 2005) and siRNAs (Chen et al., 2012) is globally reduced in cancer compared to normal cells, with ensuing alterations of the gene expression regulatory network. Exposure of cancer cells to RT inhibitors restores the normal expression profile of miRNAs, with a direct impact on global gene expression (Sciamanna et al., 2013, 2014). These lines of evidence suggest therefore that high RT expression exerts: (i) a physiological control on the biogenesis of miRNA in early embryogenesis, and (ii) a pathological role in cells conveyed toward tumorigenesis, by impairing the production of miRNAs, with the ensuing dysregulation of downstream targets and the increase of transcriptional fluctuations.

## The Remodeling of the Embryonic Epigenetic Landscape and its Implication In Evolution. A Model

In recapitulating the aspects discussed so far a framework is beginning to emerge: (1) RNA-containing nanovesicles are released from somatic tissues into the bloodstream; (2) Epididymal spermatozoa take up nanovesicles and internalize them in their nuclei; (3) The internalized RNA molecules are processed and their copy number is amplified, via the RT/DNA-dependent RNA polymerase interplay; (4) Somatic RNAs, or their cDNA copies, are delivered from sperm to embryos at fertilization.

The first three steps continuously renew the RNA storage in sperm heads. The last one, i.e., the delivery of processed somatic RNA to oocytes, can recur at each round of fertilization. Through this process sperm RNA is transmitted from one generation to the next, which can contribute to the embryo fitness and in principle expand the adaptation of the newborn to diverse environmental conditions. It is reasonable to assume that a large proportion of the males living in a same ecological niche and exposed to the same stimuli, produce sperm RNA cargos of similar composition; under constant environmental conditions, these RNA cargo would be continuously delivered to the progeny via fertilization throughout generations. It is not unreasonable to hypothesize that, in the long run (i.e., after a “sufficient” number of generations), the sperm RNAs promote the “assimilation” of new trait(s), to use Waddington’s concept (Waddington, 1959). In other words, the cumulative effects of RNA delivery through generations may promote the emergence of novel functional “canalized” pathways (Waddington, 1959), via remodeling of the embryonic chromatin architecture; in consequence, novel genetic circuits might be activated and/or pre-existing ones might be “rewired.” Mechanistically, the cumulative effects of regulatory miRNAs and tsRNAs delivered by sperm upon fertilization would drive the emergence of novel canalized pathways through two sequential steps: (i) first, by “rewiring” the expression profile of genes constituting canalized genetic circuits, and (ii) second, via targeted retrotransposition events that provide new regulatory sequences, which brings to completion the newly canalized circuits.

The first (epigenetic) step builds on the established regulatory functions of miRNAs and tsRNAs, which can modulate the expression of relevant genes. It is reasonable to hypothesize that RNAs delivered by sperm cells at fertilization also exert these regulatory functions and remodulate the gene expression profile in early embryos. Consequently, new genetic circuits (canalized circuits) become functionally active, or/and pre-existing ones are rewired.

Canalized circuits would then reach their final state through targeted retrotransposition events (genetic step). New retrotranspositions can provide additional layers of control, by inserting protein-binding sites (e.g., for transcription factors, hormones, splicing factors), as well as enhancers, promoters, insulators, etc. in new sites within the genome.

**FIGURE 1:**
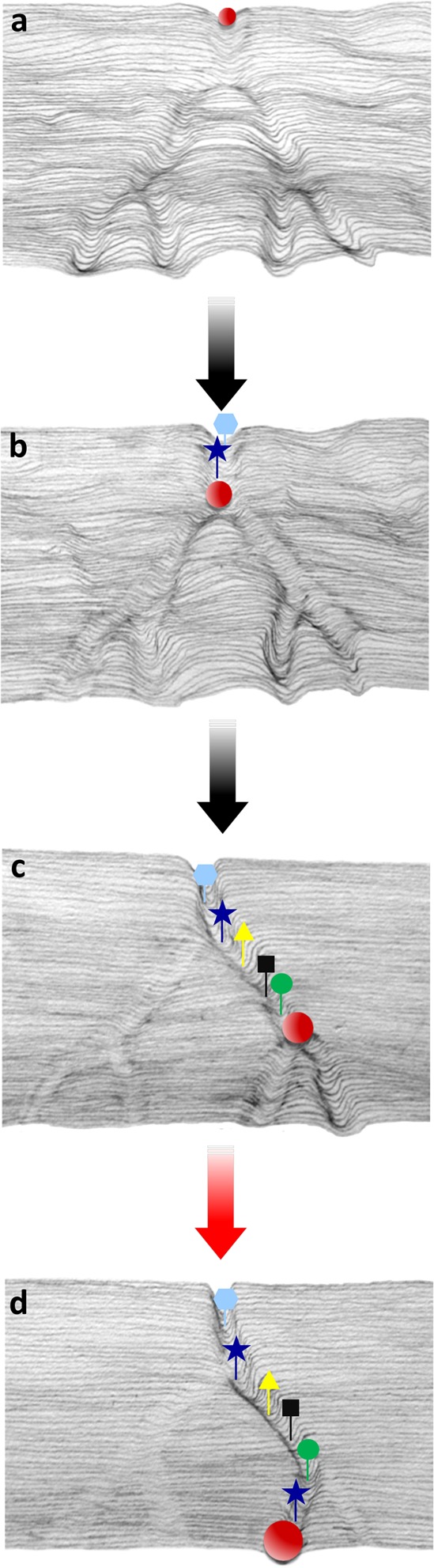
Schematic illustration, inspired by the Waddington’s model, representing the canalization process of the embryonic epigenetic landscape. The red ball rolling down from the top of the hill **(a–d)** throughout the landscape represents the preferential trajectory of the progressing epigenetic pathway driven by sperm RNAs delivered at fertilization throughout generations. Targeted retrotransposal insertions (colored shapes) follow the progressing groove **(b,c)**, and provide DNA recognition sites for gene function (transcription initiation, transcript splicing, etc.), leading to the assembly of functional regulatory networks. Insertions also favor the copy number expansion of miRNA and tsRNA coding sequences. The deeper groove with colored symbols represents the functionally active path, while the shallower grooves are not in use. The red ball in **(d)** indicates the completion of the remodeling path and the functional activation of a novel canalized pattern (red arrow).

Thus, a hybrid epigenetic/genetic process drives the remodeling of the embryonic chromatin architecture, as schematized in **Figure 1**.

The process is seen as progressive in nature, based on the assumption that the “quanta” of sperm-derived regulatory RNAs delivered to the embryo at fertilization (represented by the red ball rolling down the groove in **Figure 1**) constitute minimal contributions toward the epigenetic activation of novel canalization(s) by “deepening the canal” (**Figures 1a–c**, right branch), while non-active pathways become shallower (**Figures 1a–d**, left branch). When the cumulative effects of the sperm-delivered RNA overcome the buffering capacity of the embryos, the novel canalized genomic circuit (**Figure 1d**) has the potential to redirect the embryonic ontogenesis and generate phenotypic novelties.

Targeted retrotransposition events constitute the genetic component of the model, contributing to the functional reshaping of the embryo regulatory circuits. Targeted insertions (symbolized by different colored symbols in **Figure 1**) contribute to establish novel regulatory circuits in at least three ways: (i) they provide new protein-binding and regulatory sites; (ii) they contribute new miRNA-coding sequences that expand the overall diversity in the RNA population, and (iii) they stabilize the newly remodeled landscape by fixing the chromatin architecture.

Three considerations suggest that these changes could be permanently assimilated. First, zygotes and early embryos are thought to provide permissive, change-prone environments, consistent with the finding that the early embryonic genome is largely unstructured before zygotic genome activation, showing a low level of chromatin organization over long genomic distances (Hug et al., 2017). Second, as mentioned, LINE-1-encoded RT activity is high in preimplantation embryos (Vitullo et al., 2012) and, in parallel, the miRNA-based control system is globally suppressed (Suh et al., 2010). This is relevant in the light of evidence that miRNA-mediated control reduces random fluctuations in differentiated cells and in development, hence conferring robustness to genetic pathways (Li et al., 2009; Ebert and Sharp, 2012); on the contrary, miRNA suppression increases instability and random fluctuations in the developmental program (Hornstein and Shomron, 2006; Li et al., 2009; Ebert and Sharp, 2012). Moreover, retrotransposon families (i.e., LINE-1, Alus, LTRs) are de-repressed in embryos concomitant with global genomic hypomethylation (Lee et al., 2014; Smith et al., 2014), and constitute a potential source of both genetic and epigenetic variations (Macia et al., 2011; Vitullo et al., 2012; Fadloun et al., 2013). Overall, no massive retrotransposition events are required, with the exception of some crucial insertions that provide regulatory sequences to the newly formed canalized circuits. These crucial events would be targeted to specific hypersensitive sites generated during embryonic chromatin remodeling. Third, the RNA population that spermatozoa deliver to oocytes contains regulatory miRNAs and tsRNAs (Chen et al., 2016b), which can reshape the embryonic expression landscape and reprogram the transcription profiles of 100s of embryonic genes. Indeed, even small amounts of delivered regulatory RNAs can generate an ample spectrum of epigenetic variations with a potential impact on the phenotype. Thus, inheritable variations may be driven by small regulatory RNAs, assimilated in the change-prone genome architecture of embryos and translated into new phenotypic variants, with no major adverse effect on the permissive embryonic context.

It is worth recalling that small RNAs are involved both in macroevolutionary processes – as their number increases over time in parallel with complexity, while their loss is associated with morphological simplification (Wheeler et al., 2009; Erwin et al., 2011) – and with the canalization of genetic programs (Hornstein and Shomron, 2006; Li et al., 2009; Vidigal and Ventura, 2015).

## Conclusion

The present model of transgenerational inheritance attempts to integrate data from different sources in a biologically coherent framework. Most aspects implicated in the process are experimentally tested and are potentially able to generate transgenerationally relevant novelties. The mechanism is predominantly epigenetic and independent of genomic mutations. Importantly, the “sieving force” of natural selection is not necessary in conventional terms, because the canalization process driven by sperm RNA would generate specific pathways, leaving little space for random variations, and favoring the ultimate emergence of one or few new phenotype(s). In analogy with Lamarckism, this hypothesis is based on the assumption that extrachromosomal transgenerational inheritance can affect ontogenesis and generate evolutionarily significant, stably acquired variations. Darwinian pangenesis (Holterhoff, 2014) is the other theory with which the model has significant overlap. The hypothesis that “gemmules” containing parental characters are released from tissues and transferred to the next generation via the germline can now be reinterpreted in the light of our current knowledge of circulating nanovesicles and exosomes, carrying nucleic acids and released from somatic tissues, which can be taken up by sperm cells, thus providing a foundation for spermatozoa-mediated transgenerational inheritance. It is amazing that so-called obsolete concepts, developed in the context of two historically rejected theories, are re-emerging from modern experimental data based on next generation genomic methodologies, thus confirming that history sometime repeats itself.

## Author Contributions

CS has conceived the model and wrote the manuscript.

## Conflict of Interest Statement

The author declares that the research was conducted in the absence of any commercial or financial relationships that could be construed as a potential conflict of interest.
